# Dual-axes of functional organisation across lateral parietal cortex: the angular gyrus forms part of a multi-modal buffering system

**DOI:** 10.1007/s00429-022-02510-0

**Published:** 2022-06-07

**Authors:** Gina F. Humphreys, Roni Tibon

**Affiliations:** 1 MRC Cognition and Brain Sciences Unit, University of Cambridge, 15 Chaucer Road, Cambridge, CB2 7EF UK; 2 School of Psychology, University of Nottingham, Nottingham, UK

**Keywords:** Angular gyrus, Lateral parietal cortex, Multi-modal buffering, Temporo-spatial information, Domain-general, Connectivity

## Abstract

Decades of neuropsychological and neuroimaging evidence have implicated the lateral parietal cortex (LPC) in a myriad of cognitive domains, generating numerous influential theoretical models. However, these theories fail to explain why distinct cognitive activities appear to implicate common neural regions. Here we discuss a unifying model in which the angular gyrus forms part of a wider LPC system with a core underlying neurocomputational function; the multi-sensory buffering of spatio-temporally extended representations. We review the principles derived from computational modelling with neuroimaging task data and functional and structural connectivity measures that underpin the unified neurocomputational framework. We propose that although a variety of cognitive activities might draw on shared underlying machinery, variations in task preference across angular gyrus, and wider LPC, arise from graded changes in the underlying structural connectivity of the region to different input/output information sources. More specifically, we propose two primary axes of organisation: a dorsal–ventral axis and an anterior–posterior axis, with variations in task preference arising from underlying connectivity to different core cognitive networks (e.g. the executive, language, visual, or episodic memory networks).

## Introduction

**Fig. 1:**
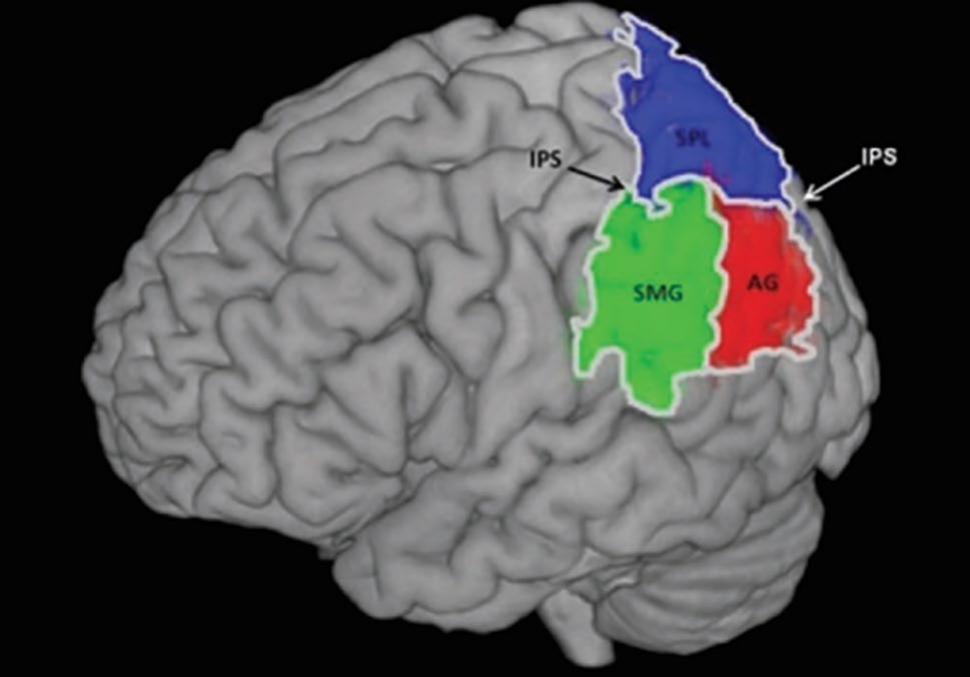
Neuroanatomical location of the parietal cortex and its major subdivisions. Here we focus on the intraparietal sulcus (IPS), angular gyrus (AG), and supramarginal gyrus (SMG)

A long history of neuropsychology and functional neuroimaging has implicated the lateral parietal cortex (LPC), including the angular gyrus (AG), in a confusing myriad of different cognitive processes and tasks, providing only a little clarity about the underlying neurocomputation. Indeed, the LPC is a heterogeneous region (see Text Box 1) with multiple graded subregions, commonly differentiated into dorsal (IPS), and ventral (AG, SMG) subregions (see Fig. 1), yet it is unclear to what extent these anatomical distinctions also indicate functional differences. To interrogate how the LPC and its subregions are implicated in different cognitive processes, two broad approaches are used in the literature. The first is a domain-specific organisation, which assumes that functions/tasks are supported by a series of discrete, neighbouring subregions (Nelson et al. 2010, 2012; Hutchinson et al. 2009; Simon et al. 2002; Seghier 2013b; Seghier et al. 2010). Prominent examples of proposed sub-regions include the intraparietal sulcus (IPS) for number processing or visuospatial tasks (Zacks 2008; Dehaene et al. 2003), supramarginal gyrus (SMG) for tool-related tasks or phonological processing (Humphreys and Ralph 2015; Hickok 2012; Ishibashi et al. 2016), and AG for episodic recollection or semantic processing (Seghier 2013b; Binder et al. 2009; Geschwind 1972; Vilberg and Rugg 2008; Shimamura 2011).

Text Box 1 As described in the main text, and depicted in Fig. 1, the LPC is segregated into two gross anatomical regions: the dorsal parietal cortex (DPC), which incorporates the intra-parietal sulcus (IPS) and the superior parietal lobule (SPL), and ventral parietal cortex (VPC), which contains the supramarginal and angular gyri (SMG and AG). These have been further subdivided via their varying cytoarchtectonic properties and structural connectivity patterns, as described in further details below (Caspers et al. 2008, 2011; Uddin et al. 2010; Cloutman et al. 2013; Mars et al. 2011). Note that, due to the considerable evolutionary expansion of human LPC (particularly the AG) detailed and precise anatomical investigation of the human LPC require studies using human participants (rather than relying on non-human animal investigations as has been used elsewhere in neuroanatomy) (Seghier 2013a; Kaas et al. 2018; Hyvärinen 1982).

A variety of anatomical techniques have been used to illustrate the structural and functional heterogeneity of the human LPC. The number of subregions identified varies depending on a variety of factors, such as the imaging modality and analytic approach. For example, using an observer-independent cytoarchtectonic analysis on 10 post-mortem brains, seven cytoarchtectonic areas in the ventral parietal cortex have been identified, two in the AG (PGa and PGp), and five in SMG (PF, PFcm, PFm, PFop, and PFt) (Caspers et al. 2008). The IPS has been similarly divided into five subdivisions in human pIPS, three on its lateral (hIP4-6) and two on its medial wall (hIP7-8) (Richter et al. 2018). Another technique involved combining diffusion-weighted magnetic resonance image (MRI)-based tractography with resting-state fMRI to derive a single LPC parcellation scheme. This technique identified 10 components (5 in the DPC and 5 in the VPC) with each subregion showing varying patterns of connectivity to the wider brain (Mars et al. 2011). In contrast, others have combined task data with resting-state functional MRI to show differential involvement of LPC subregions in distinct cognitive processes (in this case different forms of memory retrieval) (Nelson et al. 2010). In a final example, based on the compilation of the anatomy, functional connectivity, and structural connectivity of 180 cortical regions identified in the HCP, some authors have suggested a parcellation scheme of the LPC into 15 subregions, each with varying patterns of connectivity (Baker et al. 2018).

Therefore, there is clear evidence of anatomical heterogeneity across LPC. According to PUCC, this heterogeneity is functionally relevant since the ‘expressed’ contribution of each LPC subregion to different cognitive activities will be influenced by its’ connectivity with other brain regions.

The alternative, domain-general approach, suggests that at least some parietal subregions may underpin more generalised, common or “core” computations that are utilised by multiple tasks and cognitive domains. This latter approach, also known as the “primary systems hypothesis” (Ueno et al. 2011; Seidenberg and McClelland 1989; Patterson and Lambon Ralph 1999; Cabeza et al. 2018; Noonan et al. 2013; Humphreys and Lambon Ralph 2017a), observes that different cognitive processes are likely to be underpinned by combinations of more generalised neurocognitive computations and that these ‘primary systems’ will be called upon by multiple tasks (Corbetta and Shulman 2002a; Cabeza et al. 2012; Humphreys and Lambon Ralph 2015; Duncan 2010). Under this view, the common engagement of LPC regions across cognitive domains reflects the shared neurocomputation that the tasks call upon. One model that takes such a domain-global perspective is the Parietal Unified Connectivity-biased Computation (PUCC) (Humphreys et al. 2020b, 2022b; Humphreys and Lambon Ralph 2015). In the current work, we revisit this model and expand it by applying it more widely across LPC regions and multiple cognitive domains. In addition, we review recent data that relate to different aspects of this model, and outline a roadmap for future studies aiming to investigate the function of the LPC.

## Parietal unified connectivity-biased computation (PUCC) model

The Parietal Unified Connectivity-biased Computation (PUCC) takes a cross-domain perspective of the LPC function (Humphreys and Lambon Ralph 2015; Humphreys et al. 2020b). The model is based on three core assumptions. The first refers to the form of information being processed by the LPC; namely, the LPC is primarily involved in processing temporo-spatial information. Evidence suggests that there are two orthogonal forms of neural representations with differentiation across a ventral (temporal lobe) and a dorsal (parietal) pathway. Whereas the ventral processing routes generalise information across repeated episodes, leading to context-independent representations, such as those in semantic memory (Lambon Ralph et al. 2010; Lambon Ralph 2014; Buzsaki and Moser 2013), the parietal route appears to extract item-independent, temporo-spatial statistics, integrating episodes over items, resulting in representations about order, space, number, etc. (Ueno et al. 2011; Bornkessel-Schlesewsky and Schlesewsky 2013; Kravitz et al. 2011; Buckner and Carroll 2007; Pessoa et al. 2002; Humphreys and Lambon Ralph 2015).

The second assumption of PUCC is that there is a single core underlying LPC neurocomputation; online, multi-modal buffering of spatio-temporal input/output, and this function is considered to be constant across the LPC region (IPS, AG, and SMG). This kind of multi-modal convergent buffer is important to bring together temporally unfolding information from multiple different input systems to process time-extended behaviours, such as narrative speech comprehension and remembering an episodic event, or non-verbal behaviours, such as sequential object use (Botvinick and Plaut 2004, 2006; Geschwind 1965; Damasio 1989). Parallel distributed processing (PDP) models have demonstrated how this proposed underlying neurocomputation—online buffering of temporo-spatial information—can arise from the same computational process. For example, through repeated buffering of sequential input the system becomes sensitive to the regularities of sequential information (Botvinick and Plaut 2004, 2006; Ueno et al. 2011; McClelland et al. 1989). This could be likened to the formation of action/event schema, or “situation model” which specifies the temporo-spatial relationships between items in a given context.

The third key assumption of PUCC is that whilst the local neurocomputation is constant across LPC, the ‘expressed’ contribution of each LPC subregion to different cognitive activities will be influenced by its long-range connections. Thus, even on an assumption that the online buffering computation might be the same throughout the LPC, the types and forms of information being buffered will reflect the inputs and outputs to each subregion. This tenet is observed in various implemented computational models whereby a processing unit’s performance is influenced not only by its local computation but also by its connectivity to varying input/output systems (cf. ‘connectivity-constrained cognition–C^3^’: (Plaut 2002; Lambon Ralph et al. 2001; Chen et al. 2017). In terms of the underlying architecture, anatomical evidence suggests that there are variations in cytoarchitecture and functional/structural connectivity across LPC (Caspers et al. 2008, 2011; Uddin et al. 2010; Cloutman et al. 2013). Consequently, at least some domain-specific variability in the expression of cognitive function across LPC might be expected.

Therefore, according to PUCC, the emergent activation patterns across LPC results from both the core underlying neurocomputation of LPC (multi-modal buffering of temporo-spatial information) and the interaction of a region’s input and output systems, which may vary across the LPC (e.g. visual, verbal, spatial, executive, etc.) (Humphreys and Lambon Ralph 2015; Humphreys et al. 2020b).

### What is the nature of the LPC buffer?

We assume that the LPC acts as an online temporary buffer of multi-modal spatio-temporal input, rather than supporting long-term stored information per se. We propose that this buffer might be engaged by any task that involves the processing of information that is inherently varying over time and/or space. Some prominent examples are narrative comprehension or episodic recall, which require maintenance and/or manipulation of information over time. To put this in context, tasks that are less likely to require buffering, are single-item tasks that are considered more time/context independent in which the item that is currently presented does not need to be related to other internal or external forms of information. For example, picture naming tasks do not reliably engage the LPC as shown by a meta-analysis of picture-naming imaging studies (Chouinard and Goodale 2010). Notably, due to the continuous nature of our experiences, most cognitive functions would entail some level of buffering, which might be attenuated under specific conditions. In the episodic memory domain, for example, conditions that involve more buffering are those that require longer maintenance periods, integration across multiple sources, and maintenance of large amounts of information; all of these were shown to engage the AG (Ben-Zvi et al. 2015; Tibon et al. 2019; Vilberg and Rugg 2012; Guerin and Miller 2011).

Full characterisation of the temporal properties of the LPC buffering system requires additional research. In general, it has been shown that the timescales of information processing are shorter in sensory areas, and longer in “higher-order” areas (Chien and Honey 2020; Honey et al. 2012; Murray et al. 2014; Stephens et al. 2013). More specifically to the LPC, AG activation was shown to respond more strongly to information at the level of a paragraph, vs. sentences and single words (Hasson et al. 2008; Lerner et al. 2011). Furthermore, AG activation is maintained throughout the entirety of an event, whereas hippocampus activation appears to indicate event boundaries (Baldassano et al. 2017; Ben-Yakov and Henson 2018). Other functions supported by the LPC might require buffering over shorter timescales. For example, if SMG supports phonological buffering this would presumably require faster temporal resolution at the second/millisecond level. We propose that these varying timescales are one example of the functional distinctions that arise across the LPC, driven by variations in anatomical input (see below).

## Theoretical roots and current evidence of LPC “buffer”

A “buffering-type” function is consistent, and indeed partly inspired by more domain- and/or region-specific models of LPC function, such as an “episodic buffer” or a “schematic-convergence zone” in AG (Wagner et al. 2015; Shimamura 2011), or “phonological-buffer” in SMG (Baddeley 2000; Vilberg and Rugg 2008; Wagner et al. 2005), as well as a “working-memory” type system in dorsal LPC which temporarily stores and manipulates information (Pessoa et al. 2002; Humphreys and Lambon Ralph 2015). An online buffer would seem to be necessary for bottom-up capture of attention by an unexpected target (i.e., to determine what might be expected in a continuous current context (Corbetta and Shulman 2002b), for the construction of internal models, or “situation-models”, of the current world, as well as for the (re)construction of episodic and future events (Hasson et al. 2008; Lerner et al. 2011; Ramanan and Bellana 2019; Baldassano et al. 2017). Whilst domain- and/or anatomically-specific theories have been useful to account for findings from that domain of interest, they fail to explain how and why disparate cognitive domains coalesce in LPC subregions and thus what types of domain-general neurocomputation underlies processing across tasks. According to PUCC, these apparently domain-specific functions are the product of two key ingredients, namely, a common underlying temporo-spatial buffering computation combined with the varying input/output connections to other neural regions (Humphreys and Lambon Ralph 2015, 2017a; Corbetta and Shulman 2002b; Humphreys et al. 2020b).

There is a growing body of evidence in favour of a buffer-type LPC function. For instance, patient with LPC damage are not profoundly amnesic, unlike those with damage to the medial temporal lobe, rather their memory lacks clarity or vividness of episodic details (Berryhill et al. 2007; Humphreys et al. 2020b; Simons et al. 2008) which might reflect reduced ability to buffer rich representations. Furthermore, these patients demonstrate reduced ability to regenerate multi-modal associative representations (Ben-Zvi et al. 2015), as one might predict from a deficit in buffering multi-modal contextual information (Bonnici et al. 2018; Davidson et al. 2008; Yazar et al. 2014; Moscovitch et al. 2016; Shimamura 2011; St. Jacques 2019). Neuroimaging evidence supports the role of LPC in a temporo-spatial context-related processing network (although see below for important caveats when considering neuroimaging evidence of LPC function). The AG responds more strongly to images with strong rather than weak contextual associations (Bar et al. 2008), when subjects remember contextual associates of an item (Fornito et al. 2012), or when episodic memories are vividly retrieved, suggesting the recollection of rich contextually-specific details (Kuhl and Chun 2014; Tibon et al. 2019). Furthermore, it is implicated in tasks that require temporal unfolding of information, thus rely on temporal context. Some examples are episodic retrieval or future thinking (Buckner et al. 2008), narrative speech comprehension (Branzi et al. 2020), and event occurrence frequency (d’Acremont et al. 2013). The LPC is also sensitive to the temporal structure of events in linguistic, pictorial, numerical, and motoric sequence tasks (Kuperberg et al. 2003; Hoenig and Scheef 2009; Melrose et al. 2008; Tinaz et al. 2006, 2008; Gheysen et al. 2010; Stevens et al. 2005; Ciaramelli et al. 2008; Bubic et al. 2009; Humphreys et al. 2020a). We would predict that if the LPC operates as a “buffering-system” then the content represented in the system would update periodically, to reflect the changing incoming internal/external information. Indeed, a good example of evidence that the current “content” of the LPC reflects the currently processed information has been shown using MVPA in the episodic memory literature (Wagner et al. 2015; Lee and Kuhl 2016), whereby the episodic content of a person’s current recall (in this case the visual features of a face) directly aligns with decoding in the AG (Lee and Kuhl 2016).

## Anatomical evidence: variations in cytoarchitecture, structural, and functional connectivity

We assume that a wide variety of cognitive activities can arise from a single underlying neurocomputation (multi-modal buffering), with the distinctions in the expressed cognitive function arising across the region based on variations in anatomical input (e.g. visual, verbal, spatial, executive etc.) (Humphreys and Lambon Ralph 2015). In terms of the underlying architecture, anatomical evidence suggests that there are variations in cytoarchitecture and functional/structural connectivity across LPC (Caspers et al. 2008, 2011; Uddin et al. 2010; Cloutman et al. 2013) along two axes: a dorsal–ventral axis and an anterior–posterior axis. Given the anatomical heterogeneity of the LPC, at least some variability in cognitive function across LPC might be expected.

Along the dorsal–ventral axis, the dorsal LPC (dLPC) forms part of a fronto-parietal system, which is part of the multiple-demand network (Duncan 2010), whereas the ventral LPC (vLPC) connects with a distributed set of regions associated with the default mode network (DMN) or saliency network (Cloutman et al. 2013; Spreng et al. 2010; Power and Petersen 2013; Vincent et al. 2008; Uddin et al. 2010; Lee et al. 2012; Yeo et al. 2013; Power et al. 2011), with the fronto-parietal and DMN networks often showing anti-correlated activity (Keller et al. 2013; Chai et al. 2012; Fox et al. 2009; Humphreys and Lambon Ralph 2017b). The classical neuropsychological literature demonstrated that damage to dorsal vs. ventral LPC produces different patterns of deficits, such as ideomotor vs. ideational apraxia, or Bálint's vs. Gerstmann’s syndrome (Buxbaum et al. 2006; Vallar 2007). Likewise, in functional neuroimaging a dorsal–ventral difference has been observed in separate cognitive literatures. For instance, goal-directed attention vs. stimulus-driven attention, numerical calculation vs. numerical fact recall, or episodic decisions (e.g. “mnemonic accumulator”) vs. episodic recollection (Sestieri et al. 2017; Gonzalez et al. 2015; Delazer et al. 2003; Kim 2010; Vilberg and Rugg 2008; Corbetta and Shulman 2002a). In line with the assumption of the PUCC model, these task-specific variations can be explained in terms of varying input/output systems to the dLPC and vLPC. Indeed, the dLPC is structurally connected to frontal executive processing areas (Caspers et al. 2011; Cloutman et al. 2013) and forms part of a domain-general multiple-demand network, necessary for any executively demanding task (Duncan 2010). Most models of executive function or top-down attention posit a mechanism, akin to a working-memory like system, that selects and manipulates temporarily buffered information (Crowe et al. 2013). In contrast, without the direct influence of prefrontal goal-directed cognition, the vLPC will act more like a ‘slave’ buffer whereby information is accumulated and maintained throughout a sequential activity. Indeed, the divergent dorsal vs. ventral pattern is supported by evidence showing an opposing influence of task difficulty in dorsal vs. ventral LPC. Specifically, dLPC (IPS) shows a positive correlation with task difficulty across cognitive domains, whereas vLPC, which is commonly associated with the DMN, shows the inverse pattern—stronger deactivation for harder tasks (Gilbert et al. 2012; Harrison et al. 2011; Humphreys and Lambon Ralph 2017b).

In addition to the emergent dorsal–ventral connectivity/functional differences, there exists an additional anterior–posterior organisational axis within the vLPC, as evidenced by varying connectivity to the saliency network, DMN, language network, and visuospatial network (Caspers et al. 2011; Cloutman et al. 2013; Daselaar et al. 2013). These variations are reflected in differences in the emergent cognitive functions. For instance, anterior vLPC (SMG) has been associated with phonological processing and bottom-up attention, whereas the AG forms part of the DMN and is engaged by episodic/autobiographical memory retrieval, narrative comprehension, numerical fact retrieval etc. (Humphreys et al. 2020a, 2022a; Humphreys and Lambon Ralph 2015; Branzi et al. 2020; Corbetta and Shulman 2002a; Delazer et al. 2003; Kim 2010; Vilberg and Rugg 2008).

## Directly combining functional data with functional/structural connectivity

**Fig. 2:**
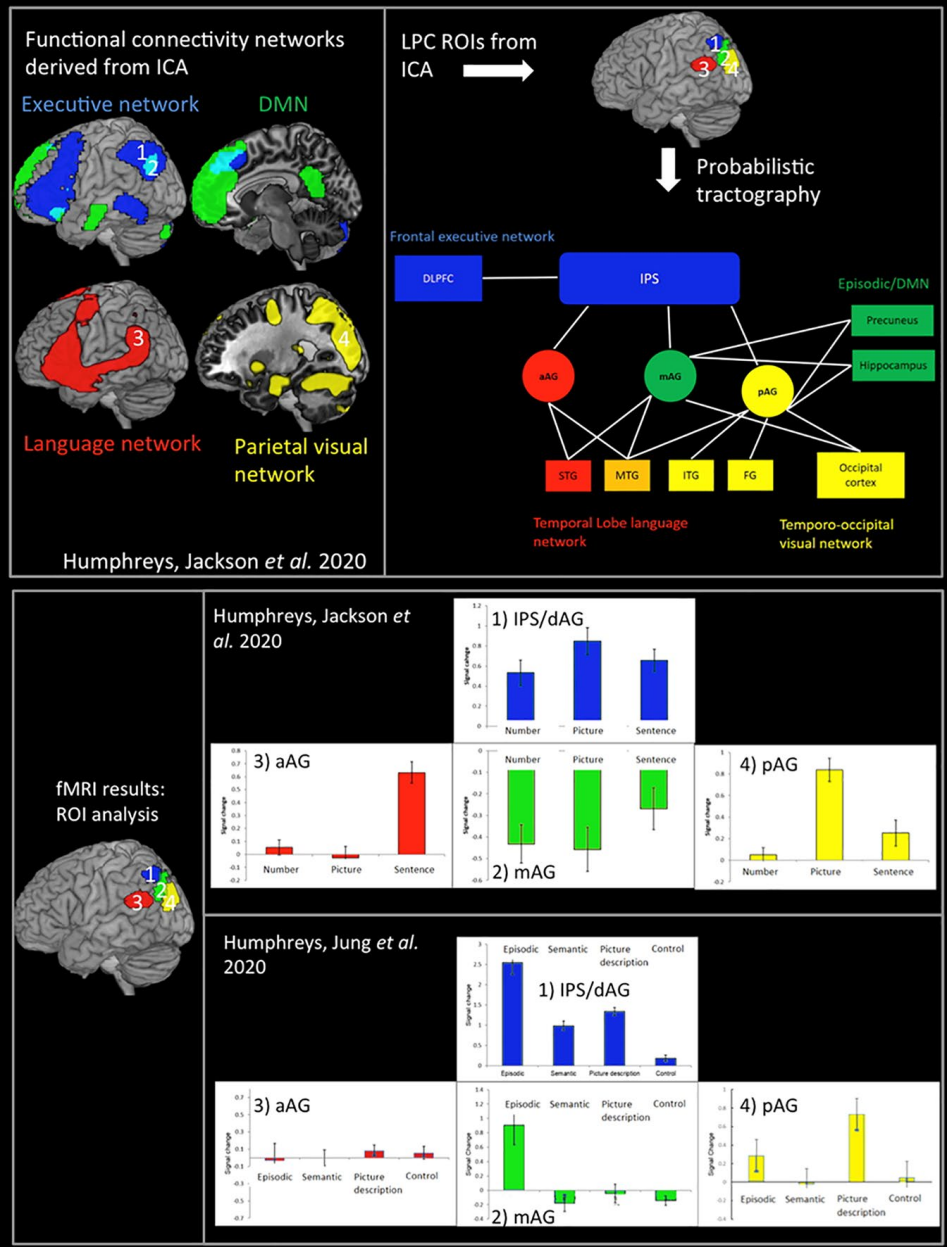
Top left: The four LPC functional connectivity networks derived from ICA (Humphreys et al. 2020a). The results show four separable functional networks (executive network in blue, DMN in green, language network in red, and parieto-visual network in yellow) that implicate different LPC regions: a dorsal region (1: dorsal PGa/IPS) and three ventral AG regions: a central region (2: mid PGp; mAG), an anterior region (3: ventral PGa; aAG), and a posterior region (4: posterior PGp; pAG). Top right: The results from the DTI analysis (Humphreys et al. 2022b), using the four functional ICA-derived ROIs as seed regions. Consistent with the functional connectivity data, the dorsal region (AG/IPS) showed long-range connectivity with lateral frontal executive control regions (dorsolateral prefrontal cortex; DLPFC). In contrast, within the ventral AG, the anterior region (aAG) showed connectivity with temporal lobe language areas (middle- and superior-temporal gyrus (MTG and STG); the mAG showed connectivity with areas involved in the DMN and the core recollection network (hippocampus and precuneus) and the pAG connected with areas including the medial parietal cortex [inferior temporal gyrus (ITG) and fusiform gyrus (FG)] and occipital cortex. Note: there are also some additional connections shown in the figure (e.g. mAG to MTG/STG, and mAG to occipital cortex), which may be consistent with the evidence of a graded- rather than sharp-shift in connectivity between regions. Bottom panel: The ROI results from two fMRI studies (Humphreys et al. 2020a, 2022b) using the same LPC regions shown in the top panel above. The task activation profile varied across subregions, the pattern of which closely mirrored its functional and structural connectivity profile. Specifically, consistent with the role of IPS as part of a domain-general executive processing network, the dorsal region (blue) demonstrated a domain-general response with the level of activation correlating with task difficulty both within and across cognitive tasks. Ventral AG also showed a functional response consistent with each region’s individual connectivity profile. Specifically, the central AG (mAG; green), which is functionally connected with the DMN and episodic retrieval network, showed a strong positive response during episodic retrieval, with activation correlating with memory vividness. This region was deactivated by all other tasks, with the level of deactivation inversely correlated with task difficulty. In contrast, the anterior region (aAG; red) that connected with the fronto-temporal language system showed positive activation for only the sentence task alone, and the posterior region (pAG; yellow) was part of the visual/SPL network only responded to tasks with pictorial stimuli (the picture sequence and picture-decision tasks)

As discussed above, the central assumptions of the PUCC model are that whilst LPC as a whole might share a common underlying neurocomputation (e.g., multimodal buffering of temporo-spatial information), variations in function across subregions arise due to differences in the underlying input (e.g. visual, verbal, spatial, executive etc.). Therefore, PUCC would predict a direct one-to-one mapping in a region’s task-related activation profile and its connectivity to certain functional networks (e.g. visual, linguistic, episodic memory etc.), with the level of activation dependent on the extent to which a particular task draws on resources from other cognitive/sensory networks. Whilst the evidence discussed above is consistent with these assumptions, a direct test of the model requires (1) within-study cross-domain comparisons, and (2) directly linking functional data with measures of functional and structural connectivity. We addressed this issue in a series of studies that especially focused on the AG and its dorsal boarder with lateral IPS (Humphreys et al. 2020a, 2022b). First, using resting-state ICA, we identified separable AG subregions, consistent with those identified elsewhere (Caspers et al. 2008, 2011; Uddin et al. 2010; Cloutman et al. 2013; Mars et al. 2011). Specifically, within the AG, we identified a dorsal region (dorsal PGa and IPS) and three ventral AG regions: an anterior region (ventral PGa), a central region (mid PGp), and a posterior region (posterior PGp). These subregions were found to have varying underlying connectivity profiles, as verified by independent DTI and resting-state fMRI analyses (see Fig. 2). Specifically, the dorsal region (AG/IPS) showed long-range connectivity with lateral frontal executive control regions in dorsolateral prefrontal cortex and inferior frontal gyrus. In contrast within vLPC, anterior AG showed connectivity with temporal lobe language areas (Lambon Ralph et al. 2017; Vigneau et al. 2006; Binder et al. 2009), the mid AG showed connectivity with areas involved in the DMN and the core recollection network (Thakral et al. 2017), including the hippocampus, as well as large portions of the precuneus (Rugg and Vilberg 2013; Sestieri et al. 2011), and the posterior AG connected with areas associated with visual processing and spatial attention, including the medial parietal cortex and occipital lobe (Zacks 2008; Corbetta and Shulman 2002b).

We then examined variations in the pattern of task-based activation across these subregions in two independent fMRI studies. In the first fMRI study, participants were presented with temporal sequences of stimuli that followed either a coherent or violated sequence in a sentence, number, and picture task (Humphreys et al. 2020a). In the second study, participants performed a series of tasks involving episodic memory retrieval, semantic memory retrieval, picture-decisions, and a low-level visual control task (Humphreys et al. 2022b). Consistent with the notion that the whole LPC is sensitive to the temporal structure of events, the results of the first study showed that all subregions within the LPC were sensitive to the coherence of the sequences. Nevertheless, the task activation profile varied across subregions, the pattern of which closely mirrored its functional and structural connectivity profile (see Fig. 2). Specifically, consistent with the role of IPS as part of a domain-general executive processing network, the dorsal region demonstrated a domain-general response with the level of activation correlating with task difficulty both within and across cognitive tasks. Ventral AG also showed a functional response consistent with each regions individual connectivity profile. Specifically, the central AG (mid PGp), which is functionally connected with the DMN and episodic retrieval network, showed a strong positive response during episodic retrieval, with activation correlating with memory vividness. This region was deactivated by all other tasks, with the level of deactivation inversely correlated with task difficulty (this result has since been replicated in a propositional speech production task (Humphreys et al. 2022a)). In contrast, the anterior region that connected with the fronto-temporal language system showed positive activation for only the sentence task, and the posterior region was part of the visual/SPL network only responded to tasks with pictorial stimuli (the picture sequence and picture-decision tasks). Interestingly, the AG showed no sensitivity to the semantic retrieval task—see below for possible explanations. Together these results fit with the PUCC model that suggests a shift in the functional engagement of vLPC based on shifts in the underlying structurally connectivity of the network.

## Important factors to consider when studying LPC function

The various studies that were conducted since PUCC was proposed, highlight some key considerations for future research. In particular, these studies demonstrate that when designing a study aiming to test the function of the LPC direct cross-domain comparisons are required. Until recently, most models have approached LPC function from a single domain (although see recent reviews Rugg and King 2018; Renoult et al. 2019). Nevertheless, reviews and cross-domain meta-analyses of existing fMRI data have clearly identified overlapping areas of activation within the LPC. These findings, however, are consistent with two alternative interpretations: (1) True overlap across tasks implicating the region in a common neurocomputation (Cabeza et al. 2012; Corbetta and Shulman 2002b; Humphreys and Lambon Ralph 2015, 2017b; Walsh 2003; Fedorenko et al. 2013) or (2) small-scale variability in function across the LPC which is blurred by cross-study comparisons or meta-analyses (Dehaene et al. 2003). Whilst highly suggestive of domain-general computations in these regions, one needs within-participant comparisons to test the model. To date, only a handful of within-study cross-domain comparisons have been conducted, and very few looked at more than two domains.

Further to this, when interpreting the results from neuroimaging studies of LPC function, it is necessary to take certain factors into account. First, the direction of activation relative to rest: given the involvement of LPC in the DMN, it is of critical importance to consider whether a task positively or negatively engages the LPC relative to rest. Contrasts between a cognitive task of interest vs. an active control condition are ambiguous because the difference could result from (1) greater positive activation for the task or (2) greater deactivation for the control. This issue becomes even more important when considering the impact of task difficulty on activation and deactivation in this region (see next). Whilst many tasks generate deactivation in the AG, this is not always the case, and the handful of activities that do positively engage the AG might be crucial sources of evidence about its true contribution (Humphreys and Lambon Ralph 2015; Humphreys et al. 2022a, 2022b). A straightforward expectation applied to almost all brain regions is that if a task critically requires the LPC then the LPC should be strongly engaged by that task. Indeed, this is the pattern observed in the anterior temporal lobe (ATL) where semantic tasks are known to positively engage the ATL relative to rest, whereas non-semantic tasks do not modulate/deactivate ATL (Humphreys et al. 2015, 2022a, 2022b). Perhaps one of the major motivations for considering task (de)activation relative to rest is that rest can be used as a common constant acting as a common reference point across tasks. This is particularly important when conducting cross-domain comparisons. For instance, when one is examining a single cognitive domain it is possible to use a within-domain baseline i.e. compare strong vs. weak demanding version of the same task (e.g. words > non-words in a semantic memory task). Since the same is not possible across cognitive domains, rest acts as a common constant for cross-domain comparisons, even if the true cognitive interpretation of rest is debated.

Second, when interpreting the results it is important to consider the impact of task-difficulty. Task-difficulty is important in multiple ways. First, it correlates positively with activation in dLPC (dorsal AG/IPS) but negatively with the level of activation within vLPC or, put in a different way, the level of deactivation in vLPC (mid-AG) is positively related to task difficulty. Indeed, the dLPC and vLPC have often been shown to be anticorrelated in resting-state data (Keller et al. 2013; Chai et al. 2012; Fox et al. 2009; Humphreys and Lambon Ralph 2017b). Furthermore, task-difficulty deactivations need to be accounted for when interpreting differences in ventral LPC areas. A ‘positive’ difference can be obtained in the AG simply by comparing easy > hard task conditions even for tasks that are entirely non-semantic, non-linguistic and non-episodic (Humphreys and Lambon Ralph 2017b). One major limitation of evidence in favour of the semantic hypothesis is that apparent semantic effects observed from fMRI studies could be explained by a difficulty confound (e.g. word > nonword, concrete > abstract). Indeed, it is known that the level of de-activation correlates with task difficulty (Gilbert et al. 2012; Harrison et al. 2011; Humphreys and Lambon Ralph 2017b)), it has been shown that one can both eliminate the difference between semantic and non-semantic tasks when task difficulty is controlled (Humphreys and Lambon Ralph 2017b) and, more compellingly, also flip the typical ‘semantic’ effects (e.g. words > nonwords, concrete > abstract) by reversing the difficulty of the tasks or stimuli (Pexman et al. 2007; Graves et al. 2017). Finally, the effect of task difficulty on LPC activation (and AG in particular) depends on specific task demands. When the AG is not critical for task performance the AG is deactivated, with stronger deactivation for hard vs. easy tasks. Note, however, that when AG is critical to task performance, such as during episodic memory retrieval, a different pattern is found: the AG is positively engaged, and increased task difficulty is associated with increased activation (Humphreys et al. 2022a, b).

A third factor that should be considered is the potential relationship between univariate activity and multivariate patterns. As abovementioned, several recent studies focusing on voxel-wise multivariate analysis of LPC regions provide evidence that the LPC holds currently processed content. For example, a study that measured brain activity during viewing and mental replay of short videos showed that univariate activity of mental replay in the dorsal AG negatively correlated with recollection, whereas the ventral and anterior parts of the AG depicted a multivariate content-sensitive signal (St-Laurent et al. 2015). In another study, participants recalled visual stimuli. Content reactivation in the LPC was then assessed via multivoxel pattern analysis, and event-specific activity patterns of recalled stimuli were found in the AG (Kuhl and Chun 2014). In a third example, information extracted from individual face images was correlated with fMRI activity patterns. In two different tasks, targeting perception and memory retrieval, the authors were able to successfully reconstruct individual faces from activity patterns within the AG (Lee and Kuhl 2016). Finally, when directly assessing the relations between pattern-based content representation and mean activation in the context of subsequent remembering (i.e., during episodic encoding) in the ventral posterior parietal cortex (vPPC), the authors showed that within the same vPPC voxels, subsequent memory was negatively predicted by mean univariate activation, but positively predicted by the strength of pattern-based information (Lee et al. 2017). These examples provide compelling evidence for stimulus-specific content representations in this region, which is consistent with our proposal of an online buffering system. They also demonstrate potential divergences between univariate and multivariate activity patterns, and that for certain cognitive functions (e.g., during memory encoding; as in Lee et al. 2017), AG might show overall deactivation, while still maintaining online information. These important variations, as well as their interactions with the other key factors mentioned above (i.e., direction of activation and task difficulty) should be carefully considered when designing experiments targeting LPC functions, and when interpreting the results thereof.

## Conclusions

Here we propose a unifying model of the lateral parietal function. We review evidence that supports the core assumptions of our proposal (although further work is needed to understand the implications of this theory; see Text Box 2 for Outstanding Questions). Namely, we propose that although a variety of cognitive activities might draw on shared underlying machinery, variations in task preference across, and wider LPC, arise from graded changes in the underlying structural connectivity of the region with different input/output information sources. More specifically, we propose two primary axes of organisation: a dorsal–ventral axis and an anterior–posterior axis, with variations in task preference arising from underlying connectivity to different core cognitive networks (e.g., the executive, language, visual, or episodic memory networks). With this, we join other theories that assign computational or process-driven rather than domain-driven roles to dissociated brain regions (Moscovitch et al. 2016).

Text Box 2 **Outstanding questions**

- What are the adaptive benefits of a multi-modal buffering system?
- How do the “executive” regions in the dorsal LPC interact with the “automated” regions in the ventral-LPC?
- Are there laterality differences in LPC engagement and/or organisation?
- How does the proposed LPC buffering system relate to other models that operationalize similar phenomena, such as the temporal context model (TCM; Howard and Kahana, 2002)

## Data Availability

Enquiries about data availability should be directed to the authors.
